# The role of community engagement in the adoption of new agricultural biotechnologies by farmers: the case of the *Africa harvest* tissue-culture banana in Kenya

**DOI:** 10.1186/s12896-017-0347-4

**Published:** 2017-03-13

**Authors:** Sunita V. S. Bandewar, Florence Wambugu, Emma Richardson, James V. Lavery

**Affiliations:** 1 McLaughlin-Rotman Centre for Global Health, Toronto, Canada; 2 Africa Harvest Biotech Foundation International Inc., Nairobi, Kenya; 3 Centre for Ethical, Social & Cultural Risk, Li Ka Shing Knowledge Institute of St. Michael’s Hospital, Toronto, Canada; 4 Clinical Epidemiology & Biostatistics Department, Faculty of Health Sciences, McMaster University, Hamilton, Canada; 5 Dalla Lana School of Public Health and Joint Centre for Bioethics, University of Toronto, Toronto, Canada

**Keywords:** Community engagement, Commercialization, Biotechnology, Agricultural biotechnology, Tissue culture, Bananas, Africa, Global health, Global development, Stakeholder engagement

## Abstract

**Background:**

The tissue culture banana (TCB) is a biotechnological agricultural innovation that has been adopted widely in commercial banana production. In 2003, Africa Harvest Biotech Foundation International (AH) initiated a TCB program that was explicitly developed for smallholder farmers in Kenya to help them adopt the TCB as a scalable agricultural business opportunity. At the heart of the challenge of encouraging more widespread adoption of the TCB is the question: what is the best way to introduce the TCB technology, and all its attendant practices and opportunities, to smallholder farmers. In essence, a challenge of community or stakeholder engagement (CE).

**Results:**

In this paper, we report the results of a case study of the CE strategies employed by AH to introduce TCB agricultural practices to small-hold farmers in Kenya, and their impact on the uptake of the TCB, and on the nature of the relationship between AH and the relevant community of farmers and other stakeholders. We identified six specific features of CE in the AH TCB project that were critical to its effectiveness: (1) adopting an empirical, “evidence-based” approach; (2) building on existing social networks; (3) facilitating farmer-to-farmer engagement; (4) focusing engagement on farmer groups; (5) strengthening relationships of trust through collaborative experiential learning; and (6) helping farmers to “learn the marketing game”. We discuss the implications of AH’s “values-based” approach to engagement, and how these guiding values functioned as “design constraints” for the key features of their CE strategy. And we highlight the importance of attention to the human dimensions of complex partnerships as a key determinant of successful CE.

**Conclusion:**

Our findings suggest new ways of conceptualizing the relationship between CE and the design and delivery of new technologies for global health and global development.

## Background

“TCB Orchard is my second husband. It fetches me money as and when I want! …I prefer TCBs because they don’t have disease, they grow faster, they give me money quicker than the traditional bananas. So I decided to plant more and more.”.[Farmer (18)].

The tissue culture banana (TCB) is a biotechnological agricultural innovation that has been adopted widely in commercial banana production. Tissue culture (TC) is based on the ability of many plant species to regenerate a whole plant from a single shoot tip. A single shoot tip is dissected and the pieces are placed in a sterile growth medium. Various hormones are added at different stages to promote shoot initiation, multiple shoot formation, and rooting induction. Within 6 months, up to 2,000 individual plantlets can be produced from a single shoot. TC technology enables rapid and large-scale proliferation of bananas. TC plants grow faster than plants grown from nursery “suckers”, are free from pests and diseases, are uniform, produce fruits earlier, and the second generation crop also matures earlier. TC bananas are ready for harvest340 days after planting, compared to 420 days for plants grown from nursery “suckers” [1].

The TCB was introduced to Kenyan farmers initially in 1997 through a partnership between the Kenyan Agricultural Research Institute (KARI) (now known as Kenya Agriculture and Livestock Research Organization) and the International Service for the Acquisition of Agri-Biotech Applications (ISAAA). The first two phases of TCB introduction were carried out between 1997–2003, during which the feasibility and appropriateness of the technology were tested and systems of production and distribution of the TCB were piloted [2]. Commercial adoption presented a challenge in Kenya because bananas have traditionally been grown by the smallholder farmers for home and local consumption [3, 4]. In 2003, Africa Harvest Biotech Foundation International (AH), a Nairobi-based non-governmental organization (NGO) that applies agricultural biotechnologies and management practices to improve the viability of smallholder agriculture [4], initiated a TCB program that was explicitly developed for smallholder farmers and adopted a ‘whole value chain’ approach that aimed to help them adopt the TCB as a scalable agricultural business opportunity [2].

Adopting TCB has been shown to increase farm and household income and reduce relative food insecurity in Kenya [2, 5]. Despite the advantages of TCB, and the importance of banana-growing in general in East Africa, TCB adoption has remained relatively low and uneven in Kenya, amounting to only 7% of total banana acreage by 2012 [3, 6]. Most TCB production takes place in 12 counties in the central and eastern provinces of Kenya, where dissemination programs, such as AH, have been concentrated [3]. One reason for the modest adoption rates of the TCB is the special agricultural management practices and higher input intensities that TCB cultivation requires. AH’s ‘whole value chain’ approach resulted from a recognition of these challenges, along with the demonstrated advantages of improved yields over a shorter growing period, a lower incidence of diseases, and greater uniformity of banana bunches, which translates into better marketability [3, 6]. At the heart of the challenge of encouraging more widespread adoption of the TCB is the question: what is the best way to introduce the TCB technology, and all its attendant practices and opportunities, to smallholder farmers. In essence, a challenge of community or stakeholder engagement (CE).

In this paper, we report the results of a case study of the CE strategies employed by AH to introduce TCB agricultural practices to small-hold farmers in Kenya, and their impact on the uptake of the TCB, and on the nature of the relationship between AH and the relevant community of farmers and other stakeholders.

CE has been recognized as an important factor in the successful introduction and adoption of new technologies [7]. But despite a growing appreciation of its importance, what makes CE effective remains poorly understood. A recent UK Parliamentary Report on lessons from the Ebola outbreak of 2015 in West Africa [8] emphasizes the need for substantial improvement to our ability to evaluate the impact of CE in global health and global development to improve effective employment of these practices.

We studied the TCB case as part of a research program on CE [9] undertaken by the Ethical, Social and Cultural (ESC) Program for the Bill and Melinda Gates Foundation’s Grand Challenges in Global Health (GCGH) initiative [10], which aimed to gain a better understanding of what makes CE effective in the context of global health and global development research. This case study was one of several that examined a CE strategy that was already considered to be successful in an attempt to describe and explain the factors that contributed to its success.

## Methods

We conducted a retrospective qualitative case study informed by grounded theory, an approach we have used successfully in other CE case studies [9, 11, 12]. The case study was conducted in collaboration with Africa Harvest (AH) in its TCB intervention areas in Murang’a County, Kenya. We made an exploratory visit to the TCB project in October 2007 to determine the scope of the case study, followed by an 8-weeks data collection visit in May-June, 2008.

We used a retrospective qualitative case study approach [13] to understand the history of the TCB Project over the previous 10 years and the role of CE practices. We conducted 16 in-depth semi-structured interviews with 17 individuals: TCB smallholder farmers (*n* = 7), AH staff (*n* = 2), field trainers/facilitators (*n* = 2); a plant biotechnology scientist from a Kenyan public agriculture research institute (*n* = 1); an agricultural NGO staff member (*n* = 1); a staff member from a private agricultural nursery (*n* = 1); a senior business consultant (*n* = 1); a consultant in community partnerships and group dynamics (*n* = 1); and a TCB extension officer from a private, farmer-owned company (*n* = 1). These interviews were complemented by non-participant observation by SB of three field training school activities at the AH TCB project intervention areas in the Thika Murang’a and Nairobi districts. Subsequently, SB conducted focus group discussions with three farmer groups at three different sites within these districts—Kairi, Muirigo and Ngorongo— on various aspects of the TCB project, with a central focus on CE activities. (Table 1) We also studied AH documents relating to TCB interventions: periodic progress reports, study reports of its formative research, training materials, and press releases and media coverage.

**Table 1:** Characteristics of focus group participants

Group name (by site)	Number of group members who participated in the focus group discussion	Gender		Age	Land ownership	No of TCB plants owned
		Women	Men			
Kairi	8	5	3	33–75	1–4 acres	10–20
Muirigo	17	5	12	39–80 years	½–5 acres with most of them with 1 acre	5–30
Ngorongo	21	3	18	30–74 years	½–10 acres with most owning 1–2 acres	3–70
Total	46	13	33			

The goal of sampling in qualitative studies is not to construct a sample that mirrors major demographic features of the target population, but rather to identify key informants with unique experiences and personal knowledge of the phenomenon in question who can provide useful descriptions, insights and explanations of events relevant to the research questions. Prospective interviewees were identified initially by AH staff members and complemented by sequential referral sampling. We approached the prospective participants at their farms or respective work places with prior appointments which were facilitated by the AH staff.

The interview guide and the consent forms were translated into Kiswahili, although many farmers in Kenya understand and speak English. The interviews were simultaneously translated by facilitators, allowing participants to switch between Kiswahili and English. Facilitators were the field trainers associated with AH, belonged to the farmers’ community, and were involved in the TCB project. Farmers’ interviews were conducted at their own farms. Interviews with other interviewees were conducted in English at their own work places. Interviews lasted between 30 and 60 min. In one instance, two farmers chose to be interviewed together. Interviews were recorded, translated and transcribed verbatim.

The analytic approach combined techniques of grounded theory and qualitative description [14–16]. Two main rationales informed our choice of method: First, grounded theory emphasizes the experiences of participants, the meaning of these experiences to participants and their understanding of events [17], as opposed to seeking confirmation of investigators’ hypotheses. Second, the grounded theory method aims to generate a theory of the phenomenon in question. The goal in this case was to produce an explanatory account that combines rich description of CE in the AH process with explanations of how various actions and structures lead to specific outcomes of interest [18].

In keeping with qualitative methodology, data collection and analysis were done in parallel [14, 19]. Preliminary data analysis was conducted by SB during the data collection phase in Nairobi to integrate initial insights into subsequent interviews. Later, upon the completion of the field work, SB conducted initial line-by-line coding of interview transcripts using the qualitative data software ATLAS.ti version 5.2., to identify key concepts, categories, and patterns. A constant comparative approach was used to compare findings within and across interviews and between categories [14, 18]. This was followed by focused coding and creation of analytical memos, as described by Charmaz [14]. This process produced a set of thematic and conceptual categories. We developed network views in ATLAS.Ti to explore relationships among the emerging concepts. Techniques for ensuring analytic rigor and trustworthiness included discussing coding between analysts (SB and JL), seeking alternative explanations for the data, and interrogating the coherence of interpretations through deliberations among the analysts [16]. We drafted a preliminary manuscript as a “best fit” for the data and refined it through several subsequent iterations.

The case study research protocol was approved by the respective Research Ethics Boards at the University of Toronto, and the Ministry of Agriculture, Nairobi, Kenya.

## Results

### The context: Introducing the TCB in the aftermath of ‘breaches of trust’

During 1970s and 1980s tea and coffee cash crops offered small-hold farmers (smallholders) in Kenya a reliable, if modest, living. Farming cooperatives offered a buffer against economic hardship, particularly for poorer farmers, and played a powerful role in shaping farming communities in the country.

“…back in the 70s and 80s…we took pride in ourselves on having been taken to school by coffee. Because, what a farmer would do if he didn’t have school fees, coffee would give him a loan to pay school fees for his children. If he didn’t have food, the coffee societies would buy maize…get food on their table… all to be deducted when farmers received their proceeds.”. [Agricultural NGO staff member (Interview # 8)].

In the early 1990s, a crash in coffee and tea prices caught smallholders off guard [20]. The economic crisis had immediate implications for the normal functioning of the tea and coffee cooperatives, as falling revenues increasingly pitted stakeholders’ interests against one another.

“There were issues of corruption…the coffee board was not operational…Farmers suffered the most since they relied so much on the coffee [plantation].”.[Agricultural NGO staff member (8)]

Inevitably, various schemes were proposed by dubious NGOs, promising farmers better markets and prices for their produce. Farmers recounted several stories of how such schemes for french beans, pineapples, avocado, maize, vanilla, agricultural water supply and collection of chameleons for export for medicinal purposes resulted in disappointment and betrayals of the farmers’ trust.

“It was bad because I remember we had planted about 2 acres of French beans…we were to harvest at least 60 cartons per day. They would come and probably [take only] 10 or 5 cartons. So we used to feed the rest to the animals…So the idea was just to sell their crops and then they go. So we have that experience, a bitter one, yes.”. [Farmers, joint interview (13)].

Struggling for subsistence, and with a reinforced fear of new initiatives and a deep distrust of ‘outsiders’, the farmers increasingly turned to bananas, known locally as a ‘back yard crop’, a ‘subsistence crop’ and a ‘woman’s crop’. Some varieties of banana, such as the imported Cavendish, proved to be viable in Kenya and offered some economic opportunity for small landholders. But infestation by pests and diseases substantially limited yields and jeopardized the viability of the banana as a cash crop [2, 21].

### Community engagement towards rebuilding ‘relationships of trust’

We identified six specific features of CE in the AH TCB project which were critical to its effectiveness.

1. Determining the local relevance of an “imported” innovation: An evidence based approach
  As part of the foundation for its CE strategy, AH chose to carry out empirical research—surveys, rapid appraisals, and qualitative studies—with farmers to gain an understanding of their perspectives and share the findings with farmers and other key constituents, such as the MoA. This approach was thought to be necessary because of the unfamiliarity of smallhold farmers with TCB. When TCB was introduced to Kenya by AH in 1997 its suitability to Kenyan climatic and soil conditions had not been tested and some preliminary field trials were conducted at Jomo Kenyatta University of Agriculture and Technology to address these gaps in knowledge. Perhaps more importantly, its acceptability to Kenyan farmers and consumers was not well established. In fact, at the outset of the program, farmers viewed the TCB as “alien” and of limited relevance as a cash-crop option.
  “Initially when we first took the plant to the field, the other farmers, and the neighbours were laughing at them and telling them what they were planting! They had never seen anything like that because a TCB plantlet looks like a small flower with just a few leaves.”. [Plant biotechnology scientist (7)]
  Understanding these initial attitudes through their empirical research helped AH to shape the content of the learning opportunities it was designing for farmers. And the discipline of seeking the perspectives of farmers also signalled AH’s desire for a respectful relationship with farmers.
  “…if your decisions are top-bottom a lot of time they either don’t work or nobody really trusts them. But most of our ideas are actually bottom-top whereby we start with the baseline. We go to the community and find out what they want…So by the time we are writing our proposal and even writing our operational strategy, we already have an idea of what people want. I think the MoA (Ministry of Agriculture) likes that, they really appreciate it.”. [AH staff (11)].
  AH’s empirical research also helped the AH team to identify community based resources, which later proved to be valuable social capital during CE: “We also wanted to find out or establish the available resources on the ground that we can work with…to identify the ground networks like community-based organizations (CBOs), field based organizations that we can work with.”. [AH staff (A)]. AH’s emphasis on generating relevant evidence also proved convincing for other key players such as the Ministry of Agriculture (MoA), which had important interests in understanding the commercial potential of the TCB.
  THE AH invested in a pilot project to assess the promise of the TCB innovation in real world context which proved critical to the TCB initiative.
  So the project went on very well and actually we proved a concept that for sure the TCB technology is workable. Farmers can adopt TCB which are actually profitable…And we found that for sure, for every one dollar you invest in the TCB, it fetches about 3 or 4 dollars back. So we were able to prove the concept…[AH staff (12)].
2. Introducing the TCB innovation: Building on existing social networks
  AH used several channels to introduce the TCB innovation, capitalizing first on those most trusted by farmers: “Because most of the time when you are approaching a community it’s usually easy when you use the existing networks than going to start afresh…Because people trust their own…[AH staff (12)]. For example, given the high levels of religious affiliation within the farming communities, AH engaged religious leaders and churches as one of their first points of contact with farmers.
  “First of all when AH came and talked to members at church some of them decided that they are going to plant the bananas. So they were picked and their names were known. Then AH requested them to go on spreading the gospel about planting the bananas.”. [Farmer (4)].
  AH leveraged the support of the Ministry of Agriculture (MoA) by sharing endorsement letters from the MoA with stakeholders during their community entry activities: “And we have the letters from the agricultural offices because that is our first point. You go through the agricultural offices to make it official.” [AH staff (B)]. This strategy helped to authenticate and legitimize AH’s messages and helped AH establish relationships with key stakeholders, such as local agricultural non-government organisations (NGOs), and various crop or livestock-based financing or management cooperatives. During our field work, we observed that farmers generally interacted comfortably and constructively with MoA staff, likely due to the positive legacy of MoA extension staff engaging with farmers throughout the country. These extension staff were also important stakeholders for AH as a result of their deep knowledge of the farming communities.
  Not all relationships between farmers and stakeholders were equally trusting. Particularly for the identification of implementation sites, AH devoted considerable time and energy to assessing levels of trust with prospective partners, and prioritized those communities where productive partnerships could be built upon a relatively trusting base.
3. Facilitating farmer-to-farmer engagement
  The TCB plantlet is vastly different from traditional bananas in its appearance. Unfamiliar appearance has been an impediment to the successful uptake of other agricultural innovations by farmers and consumers, such as with the introduction of Golden Rice in South East Asia [22] and this was true also for the TCB. But farmers’ scepticism ran deeper than the appearance of the plantlet. They were concerned, in particular, about the need for trained personnel to carefully grow TCB plantlets in nurseries, and several other unfamiliar agricultural practices that made the TCB’s commercial potential more difficult to understand.
  “…here is a technology, those are scientists and they want to reach the community and that is beyond science. So we were dealing with all the social issues…if you want to adopt a technology, it is not just about the banana, there are perceptions and attitudes… So the first thing we had to tackle, of course, was the perceptions [of farmers and communities about TCB].”. [Consultant in community partnerships and group dynamics (6)]
  AH’s claims about the viability of TCB required validation for farmers to even consider adopting the crop. AH adopted the practice of exposure visits or travelling workshops, to provide farmers with opportunities to experience TCB farming practices first-hand. ‘Exposure visits’ typically involve farmers visiting demonstration or pilot agricultural sites developed by private companies, NGOs, or government agricultural research institutes. Farmers take part in demonstrations and have an opportunity to ask questions of the organizers and peer farmers who have been early adopters of the new practices. These strategies were used by the Kenyan Ministry of Agriculture (MoA) and government institutes such as Kenya Agricultural Research Institute (KARI), to introduce new practices to Kenyan farmers.
  “So it was easier because a few farmers had walked through the whole cycle. So when it was the time for mass adoption, we gravitated around pilot sites. So that became easier because there was something they could see…just creating a desire and in the process their attitude would change. They would say ‘oh, I will try’…Exposure trips worked wonders…So, in fact, that was the most effective. So they were saying this is someone we know, it is so and so’s father but look, his is also doing well…”.[Consultant in community partnerships and group dynamics (6)]
  “So we were convinced that if those farmers are able, just the way I am talking now, I am talking from experience. They talked from experience. And having been farmers all the way through when somebody talks we could actually understand.”. [Farmers, joint interview (13)]
  Exposure visits made the potential benefits of the TCB innovation evident to interested farmers and offered them engagement with other farmers who had already adopted the TCB, sometimes from within their own social networks, offering an opportunity to assess the merits of AH’s claims. One of the farmers’ genuine concerns was about viable markets for their bananas, a concern rooted in past bitter disappointments. An AH staff member recounted the farmers’ scepticism:
  “…you have been sent from Nairobi to come and tell us to plant bananas and then after we plant, you go back to Nairobi. Where are we going to take the produce?” But we [AH team] kept on assuring them, we are with you here 3 days in a week and we will be listening to your concerns and we are going to try and see how you can work together.”. [AH staff (11)]
  One of the persistent challenges for AH was to convince farmers that bananas that had been growing effortlessly in their backyards for generations would now require more intensive and technical agronomics if they adopted TCBs. The travelling workshops and legitimate business partnerships were instrumental in guiding farmers through this transition, in stark contrast to the disreputable operators from the past.
4. Focusing engagement on farmer groups
  From the mid 1980s, contract farming evolved in Kenya to include more cooperative “schemes” whereby farmers would group together and establish their own terms of engagement with contracting companies to maintain better control of their stakes in the process [23]. These farmer groups also became eligible for various government subsidies and loan facilities, and registered groups were visited by the agriculture extension officers from the Ministry of Agriculture, who shared new information about a wide range of agricultural practices, at no cost to the individual farmers.
  AH was well aware of the advantages of farmer groups and adopted them as an explicit focus of their engagement strategy, encouraging independent farmers to consider the merits of joining with other farmers to pool risk and to better prepare for opportunities that they would be introduced to in the ‘whole value chain’ model [4].
  “Once these farmers are aware about the TC technology, about commercial banana farming, then we move on and talk about forming groups or use the existing ones which might be doing another crop, such as, avocado or maize and looking for another enterprise.”. [Field trainer/facilitator (16)]
  Professionals in the field of group dynamics and psychology from the public and private sectors, along with experts in TCB agronomy, trained the AH trainers who then engaged with farmer groups.
  “Mainly the lecturers were from outside…these trainings were very exhaustive. They handled the TCB innovation from a broad perspective…The group dynamics that we were trained in was broad…We were taught about the various methods of approaching a group. It was all broad, not specific…It was very helpful…[to learn] about group dynamics and the way groups behave.”. [Field trainer/facilitator (2)]
  A formal approach to group formation encouraged group members to develop their own “group constitutions” emphasizing transparency, equity in opportunities, and participatory decision making. These constitutions established the grounding principles for group governance. These included, among others: setting eligibility criteria for nominations for specific positions of responsibility within the group, such as chairperson, secretary, treasurer and banana grader, and procedures for matters related to finance and sales of the TCB produce.
  “It is not one person who makes decisions…We discuss the issues at hand and voice our opinions. We discuss the issues thoroughly and see what we stand to gain. …In this group we have some rules. For example, as a member of the group you cannot sell your bananas to people out there without the group deciding…You cannot as an individual farmer decide to sell yours to someone on your own. If you do so, you are asked to leave the group.”. [Ngorongo FGD] (Table 1)
  These group training activities emphasized the rationale for collective action. “You must first have a reason for forming the group. Also everyone joining the group must be interested in that reason for which you are forming the group. You must also have a vision for the future of the group for it to succeed.” [Ngorongo FGD] (Table 1). They also focused on issues of leadership within the group. Group leaders were elected by group members, which fostered a sense of legitimacy within the group, especially since they knew that they also had the power to remove leaders who were not living up to the group’s expectations. The growing collective sense of the group’s power also helped in the transition to a real sense of ownership of the TCB technology, in both a symbolic and economic sense.
  “Yes, because unless they take it as their activity, their project…we cannot say it is community development, unless they own it as their project, their bananas…So they say with or without a market, we are going to do banana farming and we are going to do it as a commercial venture. So those things I think are very important. The ownership of the project also means a lot to them.”. [Field trainer/facilitator (16)]
5. Strengthening relationships of trust through collaborative experiential learning
  Adoption of appropriate agronomy practices was crucial for the realisation of TCB’s commercial potential. Without sufficient TCB yields, AH would have run the risk of being perceived as simply another outside organization that failed farmers.
  “Once farmers planted, and when we began talking about good management and adopting a technology, that was difficult…If it was only the technology and everything else was the same it would have been easier. But now one is bringing a new technology and one is also changing the agronomy. I think that change was a lot!”. [Consultant in community work and group dynamics (6)]
  TCB farmer groups were trained in TCB-specific agronomic practices at demonstration plots, which they developed with the kinds of training described above. These plots served as ‘field schools’ for group members.
  “After forming the group, the idea [of demonstration plots] was brought by AH so that we can have a central place where we can all be coming to be taught as to how to plant…That is how we started the field school or demonstration land…Drawing upon these learning at the field school, we started doing it on our own farms where we did exactly what they had taught us…”.[Farmer (4)]
  “The AH has done an unusual thing here in the area. They have trained us on good farming practices. And they don't ask us to pay them, they pay the teacher [Trainer from the community (16)] to come and teach us. All they need is our presence to attend the training sessions. We like this aspect of them training us.” [Ngorongo FGD]
  “After a lot of training they told us to try and plant the TCB…So we saw the difference between the traditional and the TC bananas.”. [Farmer (P15)]
  The exposure visits and demonstration plots required the AH team to be in the fields with farmers in their own environment and communities. Farmers found the sustained presence of AH in their communities unusual and reassuring.
  “Because I thought for myself that they can’t work all that hard to come and visit the farmers while they are cheating…”.[Farmer (9)]
  “What made us believe is that we thought that these people cannot come all the way here to just cheat us. Because we were all grown-ups who could think. Like me personally I don’t believe someone can come from wherever to just tell you to plant something bad. So that is why I believed them.”. [Farmer (15)]
6. Learning the marketing game
  “The problems we were having before were relating to marketing. Our marketing was not that good…No customers, the bananas are ripening in the *shamba* [orchard] and trucks are not coming to collect them. There was that difficulty but nowadays not much.”. [Farmer (3)]
  The traditional bananas, particularly those of smallholders, rarely made it to the local retail market. It was imperative that farmers develop the required skills and understanding to have better control over the commercial opportunities offered by TCB. Failure to enact improvements in supply chain and marketing practices would have caused enormous damage to farmers’ trust: “Because you see they would start doubting, if you are telling us to grow bananas and if they had seen bananas rotting somewhere or bananas were being bought at 50 shillings.” [AH staff (11)]
  Because of its limited shelf-life, significant management skills are required to maintain banana quality until delivery to market. Farmers had little marketing knowledge, access to non-local markets or buffers to protect against price variations, making them vulnerable to exploitative middle men. Furthermore, public sector supports are scarce, as the banana market is largely unorganized with no government policies: “… the government actually had a very streamlined policy for marketing tea and coffee which are considered cash crops but banana has always been considered a subsistence crop which did not have this advantage.” [AH staff (12)]
  The primary goals for the commercialisation of the TCB were: (1) to develop a pool of customers with sustained interest in bulk produce; (2) consistent and timely supply of quality and quantity TCB produce to win bulk buyers’ confidence; and, consequently, (3) the establishment, over time, of a more stable demand–supply chain. For smallholders with limited financial resources, maintaining both quantity and quality of production was a formidable challenge, with many critical factors beyond their control, such as their complete reliance on rainfall for adequate irrigation.
  “Because with supermarkets, once you go into a contract with them, if they say we are going to supplying you a ton per week, it is a ton per week for 52 weeks a year! I have told you our farmers are using rain fed agriculture and under this [condition] farmers cannot achieve this [quality and quantity]…And if you are going into a contract [with bulk buyers] you must ensure that you are going to meet your part of the commitment.” [Agricultural NGO staff member (8)]
  To respond to these challenges, AH integrated marketing training for farmers in field schools, collaborating with a private company, TechnoServe, and with Tissue Culture Banana Enterprise Ltd. (TCBEL), a farmer-led marketing company whose establishment was facilitated by AH.
  “And from there I have been working as an extension officer training farmers…the post-harvest handling and organizing the market for them…So that is why I was saying that they need some retraining on the marketing…They never knew the optimum number they needed to plant and how much they can get from it…I also train them on the concept of collection centers, that is, TCB farmers have to take their produce to collection centers instead of someone to go around to individual farmers for collection…We also want to train them on financial management…”.[Field trainer/facilitator (2)]
  “TCBEL really played a very big role. So they [farmers] can now see where to sell their bananas…the market opening is a very big and very significant thing for us.”. [AH staff (12)]
  “Probably we can replicate TCBEL as a success story in other areas.”. [Agricultural NGO staff member (8)]
  AH also expanded the exposure visits to include marketing partners
  “Well, this time we had no problem because they [TechnoServe and TCBEL] came together with the AH in whom we had confidence. These two entities told us that they would take over the marketing side. They also took the trouble to take us around to other farmers where they were actually active, they were helping farmers in marketing…So we knew how they were sold. So it was something which was well arranged, I have never seen.”. [Farmers, joint interview (13)]
  TCBEL was established as a member based organisation with affordable membership fees for farmers in the neighbourhood, but has evolved to a shareholder based company run by the farmers [24]. Given the corruption-fraught history of tea and coffee cooperatives, TCBEL has set itself apart in many concrete ways, including a commitment to fair and transparent practices, such as purchasing by weight and not by bunch count, systematic paper work, periodic payment to farmers, and the complete elimination of middlemen. It has also trained staff in a wide range of skills to ensure the consistent quality of the TCB produce.
  AH’s systematic approach to TCB marketing and commercialisation has shown results. But, satisfaction with the TCB’s commercial development is uneven amongst farmers. Also, scepticism about availability of sustained markets for TCB continues.
  “But with horticulture or dairy the first market creates demand. There will be challenges. The farmers will not know how to meet the requirements of the buyers…Those problems are always there. But if there is continuity they will be handled and….So it works as long as you build trust and the moment you build trust the demand–supply chain is maintained between traders and farmers.”. [Senior Business Consultant (5)].

## Limitations

Our study has two specific limitations. First, because of its retrospective nature, participants’ accounts were vulnerable to normal limits of recall. Second, we had to rely on AH staff to help us identify participants, since there were no natural sampling frames to identify TCB farmers and external stakeholders. As a result, we might have failed to capture a fully representative range of stakeholder perspectives, including more critical and unfavourable views of AH or their CE strategies than are represented in our final sample of individual interviews and focus groups. Throughout our analyses we remained conscious of this limitation and sought to take appropriate care with the inferences we drew from our data.

## Discussion

### A values-based approach

AH’s approach to community engagement is grounded in an explicit commitment to certain core values and guiding principles. These are publicly accessible (http://africaharvest.org/) and their substance was reflected in many of the comments and actions of AH staff and other stakeholders we interviewed. These core values included a commitment to “excellence”, “institutional and scientific integrity and accountability”, “service to farm families, especially small landholders”, among others, and their guiding principles included a “commitment to partnerships that strengthen African agriculture”, a “programmatic approach based on developing the whole value chain”, “reaching out and empowering [their] stakeholders”, “ensuring gender equality and benefit sharing from the development interventions”, and “focus on impact and tangible results to the beneficiaries”, among others. This explicit values-based approach served as a public declaration by AH that it was mindful of the farmers’ previous experiences with unfair partnerships and was committed to not perpetuating these practices. Of more immediate concern for our case study, the guiding values served as *design constraints* for its CE strategy; an internally-generated framework for assessing the implications of its activities for its stakeholders. Although our findings do not permit strong conclusions about its efficacy, we believe the explicit application of values as design constraints for CE strategies is a very promising avenue for improving CE planning and management. In this case, the approach appears to have facilitated strong relationships that helped to restore farmers’ willingness to place trust in new partners, with one another, and in a novel technology and its associated agricultural practices.

### Key features of the CE strategy

Four key features of the AH’s CE approach appear to be critical to its effectiveness. First, the decision to introduce the TCB was informed by empirical evidence that was collected through explicit processes of formative research that were part of the CE strategy. Second, CE was geared towards systematically transferring skills to farmers and other relevant players, a foundational aspect of ‘technology transfer’. Exposure visits, group learning, inputs by trainers drawn from within the communities, and field schools were critical contexts for engagement among stakeholders and provided opportunities for authentic relationships to develop, which formed the basis for all the critical points of cooperation and trust that are required throughout the scale-up of the TCB operation. Third, by seeking partnerships with both public and private sector SHs, AH effectively expanded the skills, expertise and experience at its disposal to engage farmers, and help them engage with one another. In the process, it also created opportunities to enhance the capabilities of other relevant SHs, such as academic institutions, public and private research laboratories, and agricultural nurseries, while remaining front-and-centre in establishing and maintaining their trustworthiness for farmers, in particular.

Fourth, AH staff made concerted efforts to keep the promises they made to farmers. In the context of eroded trust, farmers began their journey with AH with scepticism. Kept promises—a commitment to “match words with action”—gradually won AH the farmers’ confidence. As well, the model for the TCB scale-up put decision-making in the hands of the farmers and their collectives, which represented a fundamental departure from the “old ways” and fuelled a sense of ownership of the innovation. Importantly, the AH engagement strategy required farmers to become knowledgeable and skilled through the full value chain of the TCB, breaking old patterns established by previous partners of cultivating farmers’ dependencies on them for key aspects of the production and delivery processes.

### Attention to the human dimensions of complex partnerships

Training and capacity building are typically viewed as separate categories of activity from “core” Research & Development (R&D) activities for new technologies. Although this mind-set has begun to shift [25], there are few clear examples of how training and capacity building activities have been designed and effectively integrated with the introduction of a new technology. AH’s TCB CE strategy demonstrates how training and capacity building efforts contribute directly to the development of a human infrastructure [26] to further the aims of the initiative across the TCB value chain. Given the farmers’ collective experience with exploitative partnerships in the past, AH staff understood clearly that their trustworthiness, i.e., the farmers’ willingness to place trust in them and in the technology [27] could be the difference between the success and failure of the TCB. More generally, this attention to trustworthiness requires intensive engagement and listening to stakeholders, even prior to the execution of the project, to understand where they are starting from—what experiences have they had, and how have these experiences shape their attitudes about the specific project at hand. And it requires sensitivity and thoughtful consideration of how all the actions, behaviours, and commitments the SHs will experience in the process of being “engaged” will enhance, or detract from, their confidence in placing trust in their new partners. The success of the AH TCB introduction demonstrates the critical importance of follow-through and maintaining a sustained presence in the lives of the collaborating SHs and committing to authentic relationships.

The AH results demonstrate how CE and the complex web of relationships it produces between implementers and SHs [26] can facilitate a wide range of complex interactions including extensive training, the establishment of new business partnerships and regulatory relationships. The scope of these activities/opportunities and the relationships that facilitate them, stand in contrast to the more mechanistic approaches to CE (e.g., community advisory boards (CABs)), which typically have a more remote and limited influence on the day to day details of implementation. The findings suggest that effective CE must “break the plane” of the typical advisory, or ‘message-delivery’ models of CE to create significant relationships and shared experience. It is through these that sound judgements about trustworthiness can be made, not simply through the proceedings of advisory committees and other structural mechanisms. AH offers a useful example of what is required to make this happen.

### Challenges

In cultural terms, the re-imagining of banana cultivation from a ‘back-yard’ and ‘woman’s crop’ to a scalable commercial enterprise introduces new incentives for men to ‘take over’ roles that have traditionally been performed by women. Although the creation of new economic opportunities for men is not, in itself, a threat to the well-being of women, avoidance of this type of displacement and disruption of women’s economic opportunities has been recognized by AH as a central plank in its mission and an on-going challenge. As well, there are inherent challenges for the scale-up of TCB as an economic venture, such as the heavy demand for water in an environment that is increasingly dry. Anticipating, and fairly accounting for, this type of externality is a chronic challenge for CE and fair research partnerships

## Conclusions

We undertook this case study, in part, to help explain the perceived success of AH’s CE strategy. Our findings suggest that AH’s explicit articulation of its guiding values provided critical design constraints for its CE strategy. The resulting CE efforts helped to restore and build relationships of trust between farmers, AH and a wide range of other stakeholders. AH’s attention to the interests of farmers across the ‘whole value chain’ gave rise to training and capacity building efforts that were effectively integrated into the development of the commercial enterprise. Our findings suggest new ways of conceptualizing the relationship between CE and the design and delivery of new technologies for global health and global development.

## Abbreviations

AH: Africa Harvest Biotech International Foundation
CAB: Community advisory board
CE: Community engagement
ISAAA: International Service for the Acquisition of Agri-Biotech Applications
KARI: Kenyan Agricultural Research Institute
R&D: Research & Development
TCB: Tissue culture banana
TCBEL: Tissue Culture Banana Enterprise Ltd.
